# Supplementary material for: Minimal residual ascites 3 months after TIPS implantation implicates worse clinical outcomes in patients with cirrhosis
Source: JHEP Rep. 2025 Jan 23;7(7):101335. doi: 10.1016/j.jhepr.2025.101335 (PMC12167471; doi:10.1016/j.jhepr.2025.101335)
Supplement: Multimedia component 1 [file mmc1.pdf]

Minimal residual ascites 3 months after TIPS implantation predicts worse clinical outcomes in patients with cirrhosis

Jim Benjamin Mauz, Lukas Hartl, Andrea Kornfehl, Sarah Lisa Schütte, Paul Hemetsberger, Theresa Müllner-Bucsics, Mathias Jachs, Anja Tiede, Hannah Rieland, Michael Schwarz, Nina Dominik, Georg Kramer, Bernhard Meyer, Lukas Reider, Michael Trauner, Heiner Wedemeyer, Mattias Mandorfer, Benjamin Maasoumy, Thomas Reiberger, Tammo Lambert Tergast

Table of contents

Supplementary Results……………………………………………………….……......2

Fig. S1……………………………………………………………………………….....3

Table S1…………………………………………………………………………….....4

Table S2…………………………………………………………………………….....5

Table S3………………………………………………………………………………8

Table S4…………………………………………………………………………….....9

Table S5…………………………………………………………………………….11

Supplementary Results

Patient characteristics of excluded patients (Table S1)

Table S1 depicts the key baseline characteristics of patients receiving TIPS for ascites who had to be excluded from the study (due to lack of clinical data, previous LTx, hepatic/extrahepatic malignancy, no available abdominal ultrasound at 3 months after TIPS implantation or follow-up <90 days) compared to the included population. Notably, MELD and PPG pre TIPS and relative PPG decrease did not differ between these two cohorts; while excluded patients were older and more commonly had viral etiology of cirrhosis.

Supplementary Figures

Fig. S1. Cumulative incidence of further decompensation in patients with no ascites, minimal ascites and moderate/severe ascites 3 months after TIPS implantation. Liver transplantation and death were considered as competing events. Cumulative incidences were compared using Grey's test.

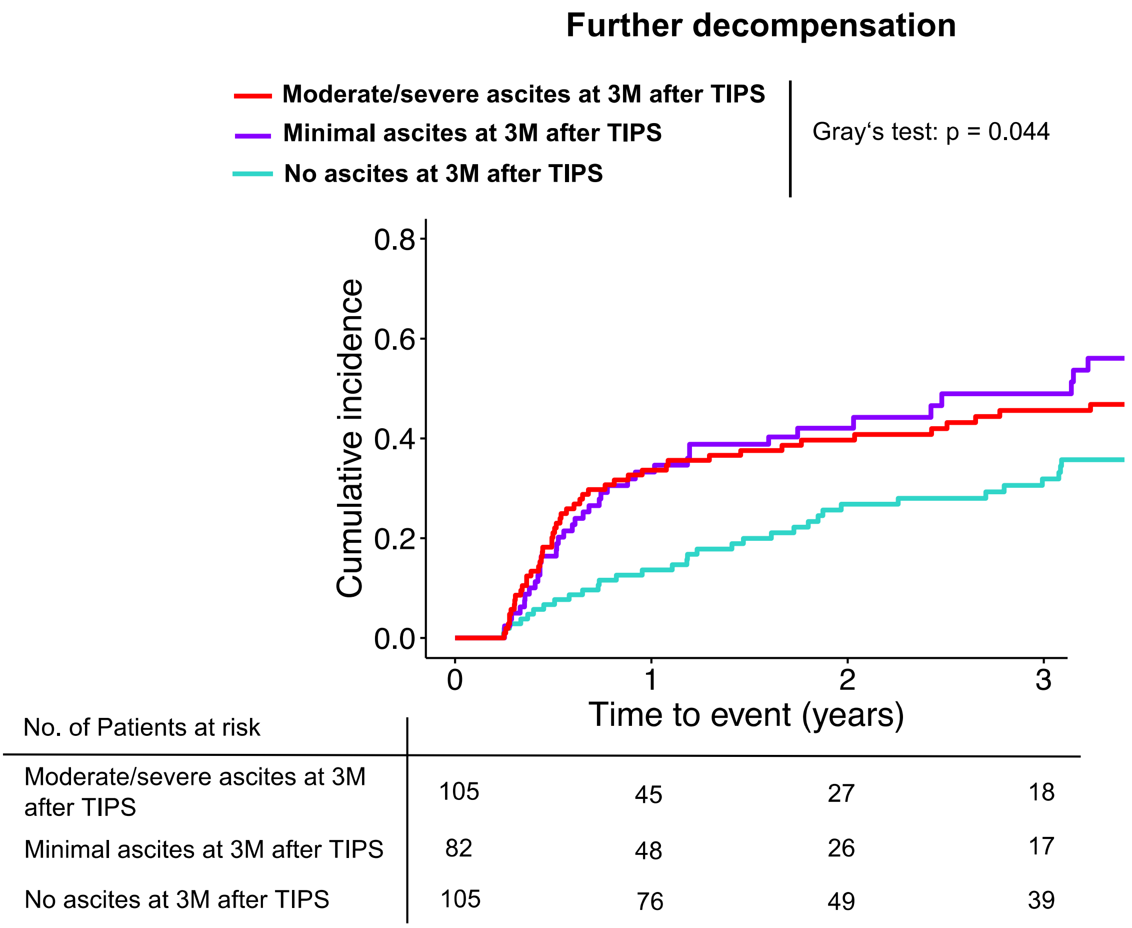

No. of Patients at risk				
Moderate/severe ascites at 3M after TIPS	105	45	27	18
Minimal ascites at 3M after TIPS	82	48	26	17
No ascites at 3M after TIPS	105	76	49	39

Abbreviations 3M: 3 months after TIPS insertion, TIPS: Transjugular intrahepatic portosystemic shunt

Supplementary Tables

Table S1. Baseline characteristics of patients who were included into the study and of patients with ACLD and TIPS implantation due to refractory ascites who were excluded due to lack of clinical data, hepatic/extrahepatic malignancy, previous liver transplantation, no available abdominal ultrasound at 3 months after TIPS implantation or follow-up <90 days.

Characteristic	Included pts n=292	Excluded pts n=331	p-value
**Sex: female,** n (%)	83 (28.4)	118 (35.6)	0.054
**Age (years),** mean ± SD	57.7 ± 10.1	59.6 ± 10.8	*0.004*
**Etiology of cirrhosis***			
**ALD,** n (%)	209 (71.6)	129 (39.0)	*<0.001*
**MASH,** n (%)	9 (3.1)	21 (6.3)	0.058
**Viral,** n (%)	29 (9.9)	122 (36.9)	*<0.001*
**Cryptogenic,** n (%)	25 (8.6)	28 (8.5)	0.964
**Other**, n (%)	20 (6.8)	31 (9.4)	0.253
**MELD score,** mean ± SD	14 ± 5	14 ± 5	0.570
**FIPS,** mean ± SD	-0.21 ± 0.78	-0.06 ± 0.93	*0.040*
**PPG pre TIPS (mmHg),** mean ± SD	18.6 ± 5.5	17.8 ± 5.8	0.074
**PPG post TIPS (mmHg),** mean ± SD	7.2 ± 3.0	6.7 ± 3.3	*0.048*
**Reduction of PPG (%),** mean ± SD	60.5 ± 15.0	61.9 ± 16.1	0.300
**Bilirubin (mg/dL),** mean ± SD	1.42 ± 1.02	1.39 ± 1.44	0.749
**Creatinine (mg/dL),** mean ± SD	1.29 ± 0.75	1.40 ± 0.93	0.096
**INR,** mean ± SD	1.28 ± 0.19	1.29 ± 0.23	0.654
**Sodium (mmol/L),** mean ± SD	134 ± 5	134 ± 6	0.393
**Albumin (g/dL),** mean ± SD	32 ± 6	30 ± 7	*0.003*

*Some patients had mixed TIPS indication and/or etiology of liver cirrhosis. Therefore, the summation of percentages may exceed 100%.

Abbreviations ALD: Alcohol-related liver disease, FIPS: Freiburg index of post-TIPS survival, INR: International normalized ratio, MASH: Metabolic dysfunction-associated steatohepatitis, MELD: Model for end-stage liver disease, PPG: Portal pressure gradient, TIPS: Transjugular intrahepatic portosystemic shunt.

Table S2. Baseline characteristics: no ascites or minimal ascites vs. moderate/severe ascites at month 3 after TIPS insertion

Characteristic	No or minimal ascites n=187 (64.0)	Moderate/severe ascites n=105 (36.0)	p-value
**Sex: female**, n (%)	63 (33.7)	20 (19.0)	*0.008*
**Age (years),** mean ± SD	57.3 ± 9.8	58.4 ± 10.6	0.373
**Etiology of cirrhosis***			
**ALD,** n (%)	127 (67.9)	82 (78.1)	0.064
**MASH,** n (%)	5 (2.7)	4 (3.8)	0.727
**Viral,** n (%)	20 (10.7)	9 (8.6)	0.560
**Cryptogenic,** n (%)	18 (9.6)	7 (6.7)	0.386
**Other,** n (%)	17 (9.1)	3 (2.9)	*0.043*
**Main indication for TIPS***			
**Ascites**, n (%)	187 (100)	105 (100)	
**PH-related Bleeding,** n (%)	18 (9.6)	6 (5.7)	0.243
**Other**, n (%)	5 (2.7)	2 (1.9)	1.000
**BL MELD score**, mean ± SD	14 ± 5	14 ± 5	0.949
**BLChild Pugh**			
**Class A,** n (%)	0 (0.0)	0 (0.0)	
**Class B,** n (%)	150 (80.2)	91 (86.7)	0.199
**Class C,** n (%)	27 (14.4)	13 (12.4)	0.624
**BL FIPS,** mean ± SD	-0.22 ± 0.77	-0.18 ± 0.78	0.645
**PPG pre TIPS (mmHg),** mean ± SD	18.9 ± 5.4	18.1 ± 5.5	0.199
**PPG post TIPS (mmHg),** mean ± SD	7.0 ± 2.9	7.6 ± 3.1	0.166
**Reduction of PPG (mmHg),** mean ± SD	12.1 ± 5.0	10.6 ± 4.6	*0.016*
**Reduction of PPG (%),** mean ± SD	62.5 ± 14.5	57.2 ± 15.2	*0.005*
**BL Bilirubin (mg/dL),** mean ± SD	1.50 ± 1.09	1.29 ± 0.87	0.104
**BL Creatinine (mg/dL),** mean ± SD	1.24 ± 0.66	1.38 ± 0.88	0.135
**BL INR,** mean ± SD	1.28 ± 0.18	1.27 ± 0.22	0.817
**BL Platelets (10^3/µL),** mean ± SD	156 ± 80	164 ± 104	0.529
**BL Sodium (mmol/L),** mean ± SD	134 ± 5	134 ± 5	*0.465*

**BL Albumin (g/dL),** mean ± SD	32 ± 6	32 ± 5	0.830
**BL AST (U/L),** mean ± SD	45 ± 27	44 ± 23	0.790
**BL ALT (U/L),** mean ± SD	28 ± 19	26 ± 14	0.365
**BL White blood cell count (10^3/µL),** mean ± SD	6.8 ± 3.0	6.2 ± 3.4	0.127
**BL Intake of diuretics,** n (%)	164 (92.1)	94 (90.4)	0.611
**Spironolactone dose (mg per day),** median (IQR)	100 (50-200)	100 (0-200)	0.768
**Furosemide dose (mg per day),** median (IQR)	60 (20-80)	60 (40-100)	0.954
**Characteristics at month 3 after TIPS insertion (3M)**			
**3M MELD score,** mean ± SD	14 ± 5	16 ± 6	*0.034*
**3M FIPS,** mean ± SD	-0.25 ± 0.8	-0.17 ± 0.8	*0.025*
**3M Bilirubin (mg/dL),** mean ± SD	2.4 ± 3.2	2.2 ± 1.9	0.427
**3M Creatinine (mg/dL),** mean ± SD	1.0 ± 0.5	1.3 ± 1.0	*0.015*
**3M INR,** mean ± SD	1.4 ± 0.3	1.4 ± 0.2	0.687
**3M Platelets (10^3/µL),** mean ± SD	139 ± 66	145 ± 87	0.511
**3M Sodium (mg/dL),** mean ± SD	136 ± 5	135 ± 5	*0.049*
**3M Albumin (g/L),** mean ± SD	31 ± 5	30 ± 6	0.081
**3M AST (U/l),** mean ± SD	53 ± 43	50 ± 45	0.587
**3M ALT (U/l),** mean ± SD	34 ± 47	25 ± 17	0.056
**3M White blood cell count (10^3/µL),** mean ± SD	7.1 ± 4.4	7.0 ± 6.0	0.913
**3M Intake of diuretics**, n (%)	153 (84.5)	86 (83.5)	0.818
**3M Spironolactone dose (mg per day),** median (IQR)	100 (38-175)	100 (0-200)	0.256
**3M Furosemide dose (mg per day),** median (IQR)	40 (0-60)	40 (0-80)	0.924

*Some patients have mixed TIPS indication and/or etiology of liver cirrhosis. Therefore, the summation of percentages may exceed 100%.

Abbreviations ALD: Alcohol-related liver disease, ALT: Alanine aminotransferase, AST: Aspartate aminotransferase, BL: at the time of TIPS insertion, FIPS: Freiburg index of post-TIPS survival, INR: International normalized ratio, MASH: Metabolic dysfunction-associated steatohepatitis, MELD: Model for end-stage liver disease, 3M: 3 Months after TIPS placement,

PH: Portal hypertension, PPG: Portal pressure gradient, TIPS: Transjugular intrahepatic portosystemic shunt.

Table S3. Incidences of events

	No ascites n=105 (36.0%)	Minimal ascites n=82 (28.1%)	Moderate/severe ascites n=105 (36.0%)
**Death**	48 (45.7%)	41 (50.0%)	59 (56.2%)
**Liver transplantation**	15 (14.3%)	7 (8.5%)	20 (19.0%)
**Further decompensation***	41 (39.0%)	40 (48.8%)	49 (46.7%)
**Worsening of Ascites#**	10 (9.5%)	22 (26.8%)	25 (23.8%)
**Overt HE**	28 (26.7%)	22 (26.8%)	24 (22.9%)
**PH-bleeding**	2 (1.9%)	0 (0.0%)	1 (1.0%)
**SBP**	2 (1.9%)	0 (0.0%)	9 (8.6%)
**HRS-AKI**	2 (1.9%)	1 (1.2%)	6 (5.7%)

*Some patients experienced multiple decompensating events at the same time. Therefore, the summation of percentages may exceed 100%.

#Worsening of ascites compatible with further hepatic decompensation was defined as formation of ascites that required paracentesis, hospitalization and/or was associated with further complications (i.e., SBP, HRS-AKI)."

Abbreviations HE: Hepatic encephalopathy, HRS-AKI: Hepatorenal syndrome - acute kidney injury, PH: Portal hypertension, SBP: Spontaneous bacterial peritonitis.

Table S4. Patient characteristics: Hannover cohort vs. Vienna cohort

**Characteristic**	**Hannover cohort n=84 (28.9%)**	**Vienna cohort n=208 (71.2%)**	**p-value**
**Sex: female,** n (%)	25 (29.8)	58 (27.9)	0.747
**Age (years),** mean ± SD	56.6 ± 11.1	58.2 ± 9.7	0.278
**Etiology of cirrhosis***			
**ALD,** n (%)	52 (61.9)	157 (75.5)	*0.020*
**MASH,** n (%)	5 (6.0)	4 (1.9)	0.126
**Viral,** n (%)	6 (7.1)	23 (11.1)	0.311
**Cryptogenic,** n (%)	9 (10.7)	16 (7.7)	0.430
**Other**, n (%)	12 (14.3)	8 (3.8)	*0.001*
**Indication for TIPS***			
**Ascites**, n (%)	84 (100)	208 (100)	
**PH-related Bleeding,** n (%)	7 (8.3)	17 (8.2)	0.964
**BL MELD score,** mean ± SD	13 ± 4	14 ± 5	*0.011*
**BL Child Pugh**			
**Class A,** n (%)	0 (0.0)	0 (0.0)	
**Class B,** n (%)	76 (90.5)	176 (84.6)	0.187
**Class C,** n (%)	8 (9.5)	32 (15.4)	0.187
**BL FIPS,** mean ± SD	-0.18 ± 0.67	-0.22 ± 0.82	0.720
**Reduction of PPG (%),** mean ± SD	60.2 ± 15.7	60.7 ± 14.7	0.818
**Bilirubin (mg/dL),** mean ± SD	1.18 ± 1.03	1.51 ± 1.00	*0.010*
**Creatinine (mg/dL),** mean ± SD	1.41 ± 0.84	1.24 ± 0.71	0.081
**INR,** mean ± SD	1.28 ± 0.19	1.27 ± 0.20	0.978
**Platelets (G/L),** mean ± SD	146 ± 94	164 ± 88	0.127
**Sodium (mmol/L),** mean ± SD	134 ± 5	134 ± 5	0.470
**Albumin (g/dL),** mean ± SD	29 ± 7	33 ± 5	*<0.001*
**AST (U/L),** mean ± SD	51 ± 29	42 ± 24	*0.015*
**ALT (U/L),** mean ± SD	30 ± 23	25 ± 5	0.069
**White blood cell count (G/L),** mean ± SD	6.2 ± 3.2	6.7 ± 3.1	0.195
**Outcomes** at 2 years post-TIPS insertion			
**Death**, n (%)	12 (14.3)	47 (22.6)	0.109
**Liver Transplantation**, n (%)	9 (10.7)	24 (11.5)	0.840
**HCC development**, n (%)	6 (7.1)	2 (1)	*0.008*
**Decompensation**, n (%)	35 (41.7)	64 (30.8)	0.075

[*]Some patients have mixed TIPS indication and/or etiology of liver cirrhosis. Therefore, the summation of percentages may exceed 100%.

Abbreviations ALD: Alcohol-related liver disease, ALT: Alanine aminotransferase, AST: Aspartate aminotransferase, BL: at the time of TIPS insertion, FIPS: Freiburg index of post-TIPS survival, HE: Hepatic encephalopathy, INR: International normalized ratio, MASH: Metabolic dysfunction-associated steatohepatitis, MELD: Model for end-stage liver disease, PH: Portal hypertension, PPG: Portal pressure gradient, TIPS: Transjugular intrahepatic portosystemic shunt, 3M: 3 Months after TIPS placement.

Table S5. Impact of minimal versus no ascites 3 months after transjugular intrahepatic portosystemic shunt (TIPS) implantation on the risk of hepatic encephalopathy. Univariable and two multivariable competing risk regression models are shown. Adjusted model I included MELD at 3M as a parameter of liver function, while Adjusted model II included FIPS at 3M. Liver transplantation and death were considered as competing risks.

Parameter of interest	Univariate (unadjusted) analysis			Adjusted model I			Adjusted model II		
hepatic encephalopathy	sHR	95%CI	p-value	asHR	95%CI	p-value	asHR	95%CI	p-value
**Minimal ascites, yes**	1.18	0.75-1.85	0.470	1.42	0.87-2.33	0.160	1.39	0.85-2.29	0.190
**Age, year**	1.02	1.00-1.05	**0.037**	1.04	1.01-1.06	**0.017**	-	-	-
**Sex (female)**	0.76	0.46-1.24	0.270	-	-	-	-	-	-
**Removed primary etiological factor at 3M, yes**	0.81	0.52-1.28	0.360	0.81	0.50-1.32	0.400	0.85	0.53-1.37	0.510
**MELD at 3M, points**	1.06	1.01-1.11	**0.014**	1.05	1.00-1.11	**0.034**	-	-	-
**FIPS at 3M, points**	1.47	1.07-2.01	**0.018**	-	-	-	1.41	1.03-1.94	**0.034**
**Albumin at 3M, g x L^{-1}**	0.96	0.92-1.00	0.060	0.99	0.94-1.03	0.570	0.98	0.93-1.02	0.290
**Sodium at 3M, mmol x L^{-1}**	0.98	0.92-1.03	0.380	-	-	-	-	-	-
**PPG reduction relative, %**	1.32	0.22-7.96	0.760	-	-	-	-	-	-
**Diuretics at 3M, yes**	1.85	0.20-17.00	0.580	-	-	-	-	-	-

Abbreviations FIPS: Freiburg index of post-TIPS survival, MELD: Model for end-stage liver disease, 3M:3 Months after TIPS placement, PPG: Portal pressure gradient, TIPS: Transjugular intrahepatic portosystemic shunt
