# Supplementary material for: Minimal residual ascites 3 months after TIPS implantation implicates worse clinical outcomes in patients with cirrhosis
Source: JHEP Rep. 2025 Jan 23;7(7):101335. doi: 10.1016/j.jhepr.2025.101335 (PMC12167471; doi:10.1016/j.jhepr.2025.101335)
Supplement: Multimedia component 4 [file mmc4.pdf]

Minimal residual ascites 3 months after TIPS implantation implicates worse clinical outcomes in patients with cirrhosis

Jim Benjamin Mauz[1,†], **Lukas Hartl**[2,3,†], Andrea Kornfehl[2], Sarah Lisa Schütte[1], Paul Hemetsberger[2], Theresa Müllner-Bucsics[2,3], Mathias Jachs[2,3], Anja Tiede[1,4], Hannah Rieland[1], Michael Schwarz[2,3], Nina Dominik[2,3], Georg Kramer[2,3], Bernhard Meyer[5], Lukas Reider[6], Michael Trauner[2], Heiner Wedemeyer[1,4], Mattias Mandorfer[2,3], Benjamin Maasoumy[1,4], Thomas Reiberger[2,3,7,*,‡], Tammo Lambert Tergast[1,2,3,‡]

JHEP Reports **2025**. vol. 7 | 1–9

Check for updates

Background & Aims: Transjugular intrahepatic portosystemic shunt (TIPS) implantation is indicated for recurrent/refractory ascites in patients with cirrhosis. The prognostic impact of residual minimal ascites after TIPS implantation has not yet been investigated.

Methods: We included patients with cirrhosis undergoing covered TIPS implantation for refractory ascites in Vienna (2000–2022) and Hannover (2009–2021) with available abdominal ultrasound 3 months after TIPS insertion (3M). The patients were followed up for further decompensation and transplant-free mortality. Two distinct competing risk regression models (Adjusted model I and Adjusted model II) were performed to determine the prognostic impact of no *vs.* minimal ascites at 3M.

Results: Overall, 292 patients with male predominance (71.7%) and mostly alcohol-related liver disease (71.7%) were included. At 3M, n = 105 (36.0%) patients showed no ascites on abdominal ultrasound, whereas n = 82 (28.1%) exhibited minimal and n = 105 (36.0%) moderate/severe ascites. The portal pressure gradient after TIPS implantation was similar in the three groups (median 7 mmHg; $p = 0.311$). Patients with no or minimal ascites had comparable Model for End-Stage Liver Disease and Freiburg Index of Post-TIPS Survival scores at baseline and 3M. Competing risk regression models showed that minimal ascites (*vs.* no ascites) was an independent predictor of further decompensation (Adjusted model I: adjusted subdistribution hazard ratio [aSHR], 1.69; 95% CI, 1.03–2.77; $p = 0.038$; Adjusted model II: aSHR, 1.76; 95% CI, 1.07–2.88; $p = 0.026$) and transplant-free mortality (Adjusted model I: aSHR, 1.76; 95% CI, 1.08–2.88; $p = 0.024$; Adjusted model II: aSHR, 1.73; 95% CI, 1.05–2.82; $p = 0.030$).

Conclusions: Patients with residual minimal ascites at 3M remain at higher risk for further decompensation and transplant-free mortality compared with those with no residual ascites.

© 2025 The Author(s). Published by Elsevier B.V. on behalf of European Association for the Study of the Liver (EASL). This is an open access article under the CC BY license (http://creativecommons.org/licenses/by/4.0/).

Introduction

The onset of ascites is the most frequent index decompensation in patients with advanced chronic liver disease (ACLD) and portal hypertension (PH).[1,2] Large-volume paracentesis, albumin infusion, and diuretic therapy can often achieve symptom control in these patients.[3] Furthermore, treating the underlying cause of liver injury can prevent further decompensation and could also lead to regression of the disease state and recompensation.[4,5] However, some patients develop recurrent or even refractory ascites.[6,7] In this setting, quality of life and prognosis are drastically compromised.[8] The introduction of the transjugular intrahepatic portosystemic shunt (TIPS) revolutionized the treatment of PH-derived complications.[7] TIPS insertion leads to a considerable decrease in the portal pressure gradient (PPG), one of the key drivers behind many complications in ACLD, thereby leading to ascites resolution in many patients and an overall improved survival.[9]

Even though TIPS implantation is effective in the treatment of recurrent or refractory ascites, some patients still suffer from clinically overt or minimal ascites months after TIPS implantation.[9,10] Notably, most decompensations after TIPS implantation occur within the first 3 months after implantation.[11] Grade II (moderate) or Grade III (severe) ascites lead to direct symptoms and often have therapeutic consequences. However, Grade I (minimal) ascites can only be detected via imaging.[12] Here, the therapeutic or prognostic consequences remain a matter of debate.[13] Although there is at least some evidence that suggests that clinically overt ascites is associated with an elevated risk for clinical complications and death, there are insufficient data regarding the clinical relevance of minimal residual ascites after TIPS implantation.[14]

Hence, this study investigated the prognostic relevance of the persistence of ascites 3 months after TIPS insertion (3M) stratified for no, minimal, and moderate/severe ascites.

* Corresponding author. Address: Division of Gastroenterology and Hepatology, Department of Internal Medicine III, Waehringer Guertel 18-20, A-1090 Vienna, Austria. Tel.: +43 1404 006 5890, fax: +43 1404 004 7350.
E-mail address: thomas.reiberger@meduniwien.ac.at (T. Reiberger).
† These authors contributed equally and share first authorship.
‡ These authors contributed equally and share senior authorship.
https://doi.org/10.1016/j.jhepr.2025.101335

Material and methods

Patient cohort

All patients with ACLD who received a polytetrafluoroethylene (PTFE)-covered TIPS for the treatment of refractory ascites during the study period in the respective centers were evaluated retrospectively for this study. We included patients who underwent TIPS implantation at Hannover Medical School during 2009–2021 (Hannover cohort), and patients who underwent TIPS placement at the Medical University Vienna during 2000–2022 or at Klinik Favoriten during 2000–2018 (Vienna cohort). Only patients with available follow-up and abdominal ultrasound at 3M were included in this study. We excluded patients with active malignancy, hepatocellular carcinoma, previous liver transplantation, or an insufficient data set. After considerate application of the selection criteria, 292 patients remained eligible for further analysis (Fig. 1).

TIPS placement

TIPS placement was performed by experienced radiologists according to the respective institutional standard operating procedures. Stents (Viatorr®; Gore, Flagstaff, AZ, USA) with a diameter of 6 mm, 8–10 mm, or 12 mm were used in all cases.

Data collection and definitions

Patients' characteristics and clinical endpoints were assessed via careful evaluation of the patients' medical records. Ascites severity was determined based on abdominal ultrasound at 3M, which was routinely scheduled in all patients undergoing TIPS placement for ascites and classified according to current EASL guidelines.[12] Patients were followed up for the incidence of clinical endpoints, including death, liver transplantation (LTx), and further decompensation. Further decompensation was modeled on the definitions outlined in the Baveno VII consensus as worsening of ascites, hepatic encephalopathy (HE), portal hypertensive bleeding, spontaneous bacterial peritonitis (SBP) or hepatorenal syndrome–acute kidney injury (HRS-AKI).[7] Worsening of ascites compatible with further hepatic decompensation was defined as the formation of ascites that required paracentesis, hospitalization, and/or was associated with further complications (i.e., SBP or HRS-AKI). TIPS implantation was considered to be a recompensating event. Only the first occurring decompensation was recorded.

Study design

The endpoints of this study were: (1) LTx-free survival as the primary outcome and (2) the incidence of further decompensation following the 3M ultrasound as secondary outcome. To investigate the relevance of different grades of residual ascites

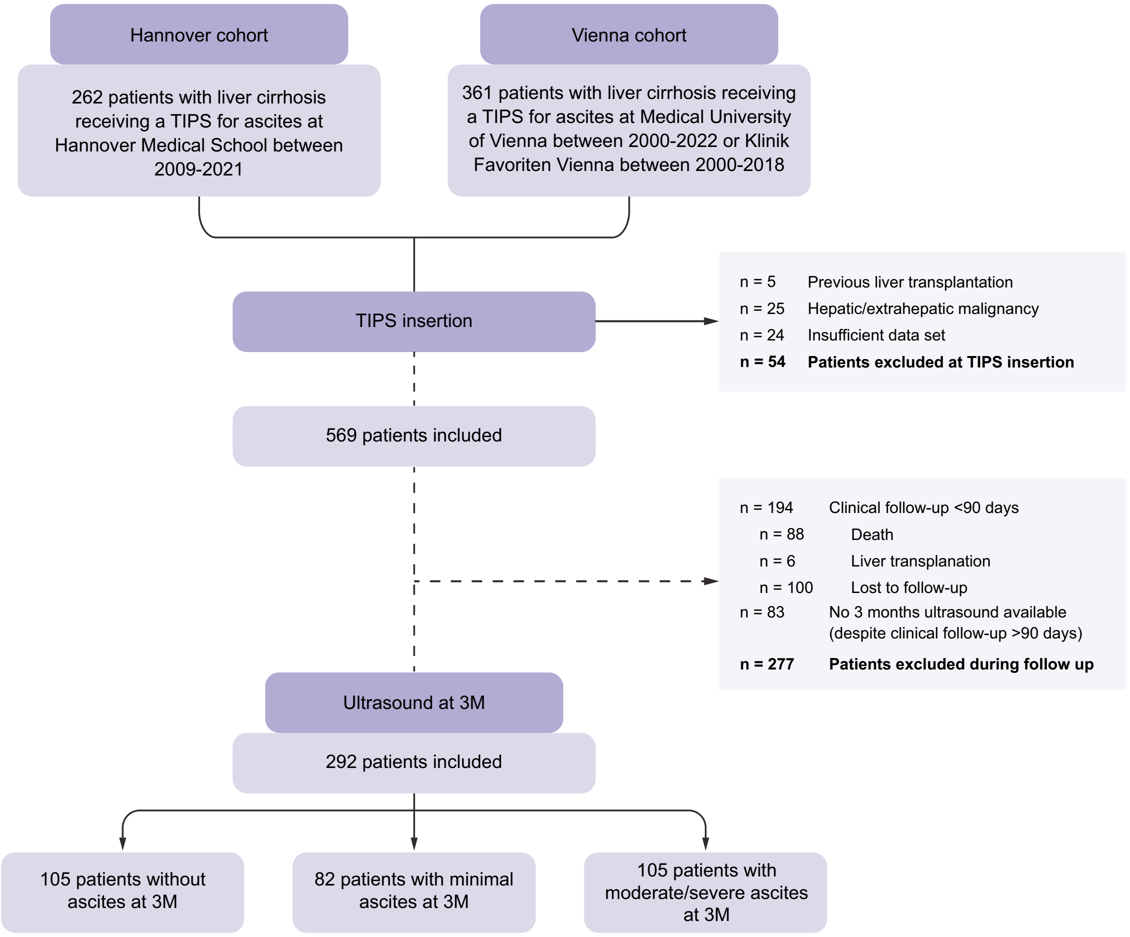

Fig. 1. Patient selection and exclusion criteria. 3M, 3 months after TIPS insertion; TIPS, transjugular intrahepatic portosystemic shunt.

at 3M, we conducted two different analyses: (1) Analysis 1: the impact of no ascites *vs.* minimal ascites *vs.* moderate/severe ascites at 3M; and (2) Analysis 2: the impact of minimal ascites compared with no ascites at 3M.

Statistical analysis

All statistical analyses and data visualizations were performed utilizing IBM SPSS Statistics (Version 28, SPSS Inc., Chicago, IL, USA) and R 4.2.1 (R Core Team, R Foundation for Statistical Computing, Vienna, Austria). Categorial variables are summarized as n (%) and compared using the chi-squared or Fisher's exact testing as appropriate. Continuous variables are displayed as mean ± SD and compared using Mann-Whitney U-test or an unpaired *t* test as required. For comparing continuous variables between the three groups, either a one-way analysis of variance or Kruskal-Wallis tests was performed, as appropriate. Clinical outcomes of patients with no ascites, minimal ascites, and moderate/severe ascites at 3M were compared using cumulative incidence calculations. Gray's test, as previously described, was computed for cumulative incidence comparison.[15] Two different Fine and Gray competing risk regression models using the R package cmprsk were calculated to evaluate whether the presence of minimal ascites at 3M compared with no ascites was associated with the risk of clinical events of interest.[15,16] Apart from minimal *vs.* no ascites at 3M, and parameters at least tendentially associated with the outcome of interest in univariate analysis (*i.e.* p <0.100), well-established and predefined parameters impacting clinical outcomes in ACLD (removed primary etiological factor at 3M, as well as parameters of liver dysfunction, *i.e.* Adjusted model I: Model for End-Stage Liver Disease [MELD] score at 3M; Adjusted model II: Freiburg index for post-TIPS survival [FIPS] at 3M) were evaluated by multivariate analysis. LTx and death (where appropriate) were considered as competing risks for both cumulative incidence comparison and competing risk regression. Only events occurring after 90 days of follow-up (*i.e.* after ultrasound at 3M) were considered for cumulative incidence comparison and competing risk regression.

Throughout the study, a two-sided p <0.05 was acknowledged to determine statistical significance.

Ethics

Retrospective analysis was approved by the Ethics Committees at Hannover Medical School (Nr. 10073 BO K 202), the Medical University of Vienna (EK 1760/2014, EK 1943/2017), and the City of Vienna (MA15, 14-264-VK). The need for signed informed consent was waived because of the retrospective study design. This study fully complies with the principles stated by the current Declaration of Helsinki.

Results

Patient selection

Initially, 623 patients with liver cirrhosis received a TIPS for ascites at Hannover Medical School (2009–2021) and the Medial University Vienna cohort (2000–2022) (Fig. 1). At the time of TIPS placement, 54 patients were excluded because of previous LTx, hepatic/extrahepatic malignancy, or an insufficient clinical data set. Furthermore, during the 90 days following TIPS insertion, 277 patients had to be excluded because of insufficient clinical follow-up (88 patients died, six underwent LTx, 100 were lost to follow-up, and 83 patients did not have 3M ultrasound available despite their clinical follow-up exceeding 90 days).

Patient characteristics

In total, 292 patients with ACLD and TIPS implantation were included in our study (Tables 1 and 2). Most of the patients included were male (71.6%); the mean age at TIPS insertion was 57.7 years. Alcohol-related liver disease (ALD) was the leading cause of cirrhosis in the cohort (71.6%), followed by viral (9.9%) and cryptogenic etiology (8.6%). All included patients received their TIPS primarily for refractory ascites and 24 (8.2%) patients additionally had a history of PH-related bleeding. At 3M, 105 (36.0%) patients did not show ascites on ultrasound, whereas 82 (28.1%) had minimal ascites and 105 (36.0%) displayed moderate/severe ascites. Overall, 136 patients required paracentesis within 90 days after TIPS implantation, most of whom displayed moderate/severe ascites at the 3M ultrasound (n = 81/105 [77.1%] *vs.* no ascites: n = 24/105 [22.9%] *vs.* minimal ascites: n = 31/82 [37.8%]; p <0.001). Details regarding the characteristics of patients who underwent TIPS placement for ascites but were excluded from the study are provided in the supplementary data online (Table S1). The supplementary data online also contains the comparison between the patient characteristics of the Hannover cohort and Vienna cohort (Table S4).

Characteristics of patients with no ascites *vs.* minimal ascites *vs.* moderate/severe ascites at 3M

Table 1 and Table S2 compare the characteristics of patients with no *vs.* minimal *vs.* moderate/severe ascites at 3M, respectively.

Patients who displayed moderate/severe ascites at 3M were more frequently male (no ascites, 63.8%; minimal ascites, 69.5%; moderate/severe ascites, 81.0%; p = 0.020). The etiology of liver cirrhosis was comparable between all three groups. Even though markers of disease severity and mortality (MELD, Child Pugh Class, and FIPS) were initially balanced between the groups at the time of TIPS placement (MELD: no ascites, 14 *vs.* minimal ascites, 14 *vs.* moderate/severe ascites, 14; p = 0.868; Child Pugh Class B: 84.8% *vs.* 86.6% *vs.* 87.6%; p = 0.831; FIPS: -0.3 *vs.* -0.13 *vs.* 0.18; p = 0.330), MELD and FIPS were both numerically higher in the moderate/severe ascites group at 3M (3M MELD: no ascites, 14 *vs.* minimal ascites, 14 *vs.* moderate/severe ascites 16; p = 0.090; 3M FIPS: -0.24 *vs.* -0.26 *vs.* -0.17; p = 0.082). Mean relative PPG reduction was significantly lower in the moderate/severe ascites group compared with the minimal or no ascites group (no ascites, 62.0% ± 14.8%; minimal ascites, 63.2% ± 14.1%; moderate/severe ascites, 57.2% ± 15.2%; p = 0.017), even though the mean absolute PPG after TIPS implantation was comparable between the groups (no ascites, 7.2 mmHg; minimal ascites, 6.9 mmHg; moderate/severe ascites, 7.6 mmHg; p = 0.311). Notably, neither the intake nor the mean dose of diuretics were significantly different at 3M between patients with no ascites, minimal ascites, or moderate/severe ascites.

Table 1. Patient characteristics: no ascites *vs.* minimal ascites *vs.* moderate/severe ascites at 3 months after transjugular intrahepatic portosystemic shunt placement.

Characteristic	No ascites n = 105 (36.0%)	Minimal ascites n = 82 (28.1%)	Moderate/severe ascites n = 105 (36.0%)	*p* value
Sex: female, n (%)	38 (36.2)	25 (30.5)	20 (19.0)	0.020
Age (years), mean ± SD	57.1 ± 9.9	57.7 ± 9.7	58.4 ± 10.6	0.617
Etiology of cirrhosis*				
ALD, n (%)	71 (67.6)	56 (68.3)	82 (78.1)	0.179
MASH, n (%)	3 (2.9)	2 (2.4)	4 (3.8)	0.853
Viral, n (%)	15 (14.3)	5 (6.1)	9 (8.6)	0.150
Cryptogenic, n (%)	8 (7.6)	10 (12.2)	7 (6.7)	0.371
Other, n (%)	8 (7.6)	9 (11.0)	3 (2.9)	0.086
Indication for TIPS*				
Ascites, n (%)	105 (100)	82 (100)	105 (100)	—
PH-related bleeding, n (%)	12 (11.4)	6 (7.3)	6 (5.7)	0.302
Other, n (%)	1 (1.0)	4 (4.9)	2 (1.9)	0.202
PPG pre TIPS (mmHg), mean ± SD	19.1 ± 5.2	18.7 ± 5.6	18.1 ± 5.5	0.370
PPG post TIPS (mmHg), mean ± SD	7.2 ± 2.7	6.9 ± 3.2	7.6 ± 3.1	0.311
Reduction of PPG (mmHg), mean ± SD	12.2 ± 4.9	11.9 ± 5.1	10.6 ± 4.6	0.053
Reduction of PPG (%), mean ± SD	62.0 ± 14.8	63.2 ± 14.1	57.2 ± 15.2	0.017
Characteristics at TIPS insertion (BL)				
BL MELD score, mean ± SD	14 ± 5	14 ± 5	14 ± 5	0.868
BL Child Pugh				
Class A, n (%)	0 (0.0)	0 (0.0)	0 (0.0)	—
Class B, n (%)	89 (84.8)	71 (86.6)	92 (87.6)	0.831
Class C, n (%)	16 (15.2)	11 (13.4)	13 (12.4)	0.831
Previous HE, n (%)	21 (20.0)	12 (14.6)	21 (20.0)	0.569
BL FIPS, mean ± SD	-0.30 ± 0.82	-0.13 ± 0.71	-0.18 ± 0.78	0.330
BL bilirubin (mg/dl), mean ± SD	1.45 ± 0.98	1.55 ± 1.22	1.29 ± 0.87	0.212
BL creatinine (mg/dl), mean ± SD	1.22 ± 0.66	1.26 ± 0.67	1.38 ± 0.88	0.313
BL INR, mean ± SD	1.27 ± 0.18	1.28 ± 0.19	1.27 ± 0.22	0.912
BL platelets (G/L), mean ± SD	149 ± 72	166 ± 90	164 ± 104	0.272
BL sodium (mmol/L), mean ± SD	135 ± 4	133 ± 5	134 ± 5	0.046
BL albumin (g/dl), mean ± SD	32 ± 6	31 ± 7	32 ± 5	0.441
BL AST (U/L), mean ± SD	42 ± 23	49 ± 31	44 ± 23	0.257
BL ALT (U/L), mean ± SD	26 ± 16	30 ± 22	26 ± 14	0.358
BL white blood cell count (G/L), mean ± SD	6.7 ± 2.8	7.0 ± 3.3	6.2 ± 3.4	0.265
BL intake of diuretics, n (%)	87 (88.8)	77 (96.3)	94 (90.4)	0.181
Spironolactone dose (mg per day), median (IQR)	150 (50–200)	100 (50–200)	100 (0–200)	0.506
Furosemide dose (mg per day), median (IQR)	40 (6–80)	80 (40–120)	60 (40–100)	*0.025*
**Characteristics at 3M**				
3M MELD score, mean ± SD	14 ± 5	14 ± 5	16 ± 6	0.090
3M FIPS, mean ± SD	-0.24 ± 0.8	-0.26 ± 0.8	-0.17 ± 0.8	0.082
3M bilirubin (mg/dl), mean ± SD	2.4 ± 3.0	2.5 ± 3.3	2.2 ± 1.9	0.728
3M creatinine (mg/dl), mean ± SD	1.0 ± 0.5	1.1 ± 0.5	1.3 ± 1.0	*0.043*
3M INR, mean ± SD	1.3 ± 0.2	1.4 ± 0.4	1.4 ± 0.2	0.478
3M platelets ($10^3/\mu l$), mean ± SD	132 ± 56	147 ± 77	145 ± 87	0.221
3M sodium (mg/dl), mean ± SD	137 ± 4	136 ± 5	135 ± 5	0.098
3M albumin (g/L), mean ± SD	32 ± 5	30 ± 5	30 ± 6	*0.024*
3M AST (U/L), mean ± SD	57 ± 50	49 ± 31	50 ± 45	0.478
3M ALT (U/L), mean ± SD	33 ± 22	37 ± 66	25 ± 17	0.120
3M white blood cell count ($10^3/\mu l$), mean ± SD	7.1 ± 4.1	7.1 ± 4.9	7.0 ± 6.0	0.994
3M intake of diuretics, n (%)	88 (87.1)	65 (81.1)	86 (83.5)	0.546
Spironolactone dose (mg per day), median (IQR)	100 (50–150)	100 (0–188)	100 (0–200)	0.489
Furosemide dose (mg per day), median (IQR)	20 (0–40)	40 (0–80)	40 (0–80)	0.246

Categorial variables were compared using the chi-squared test or Fisher's exact test, as appropriate. Continuous variables were compared using one-way analysis of variance or Kruskal-Wallis test, as appropriate.
Therefore, the summation of percentages may exceed 100%. 3M, 3 months after TIPS placement; ALD, alcohol-related liver disease; ALT, alanine aminotransferase; AST, aspartate aminotransferase; BL, baseline at the time of TIPS insertion; FIPS, Freiburg Index of Post-TIPS Survival; HE, hepatic encephalopathy; INR, international normalized ratio; MASH, metabolic dysfunction-associated steatohepatitis; MELD, Model for End-Stage Liver Disease; PH, portal hypertension; PPG, portal pressure gradient; TIPS, transjugular intrahepatic portosystemic shunt.
*Some patients have mixed TIPS indication and/or etiology of liver cirrhosis.

Characteristics of patients with no *vs.* minimal ascites at 3M

Patient characteristics, including parameters of liver dysfunction (MELD score, FIPS, and albumin) did not differ in patients with no *vs.* minimal ascites at 3M after TIPS implantation (Tables 1 and 2). Moreover, patients with no *vs.* minimal ascites at 3M after TIPS implantation were of a similar age, PH severity (i.e., PPG before and after TIPS), and etiology of liver disease. Similarly, there were no differences in the intake of diuretic

Table 2. Patient characteristics: no ascites *vs.* minimal ascites at 3 months after transjugular intrahepatic portosystemic shunt (Analysis 2).

Characteristic	No ascites n = 105 (56.1%)	Minimal ascites n = 82 (43.9%)	*p* value
Sex: female, n (%)	38 (36.2)	25 (30.5)	0.413
Age (years), mean ± SD	57.1 ± 9.9	57.7 ± 9.7	0.668
Etiology of cirrhosis*			
ALD, n (%)	71 (67.6)	56 (68.3)	0.922
MASH, n (%)	3 (2.9)	2 (2.4)	1.000
Viral, n (%)	15 (14.3)	5 (6.1)	0.072
Cryptogenic, n (%)	8 (7.6)	10 (12.2)	0.292
Other, n (%)	8 (7.6)	9 (11.0)	0.428
Main indication for TIPS*			
Ascites, n (%)	105 (100)	82 (100)	—
PH-related bleeding, n (%)	12 (11.4)	6 (7.3)	0.344
Other, n (%)	1 (1.0)	4 (4.9)	0.170
BL MELD score, mean ± SD	14 ± 5	14 ± 5	0.594
BL Child Pugh			
Class A, n (%)	0 (0.0)	0 (0.0)	—
Class B, n (%)	80 (76.2)	70 (85.4)	0.118
Class C, n (%)	16 (15.2)	11 (13.4)	0.725
BL FIPS, mean ± SD	-0.30 ± 0.82	-0.13 ± 0.71	0.156
PPG pre TIPS (mmHg), mean ± SD	19.1 ± 5.2	18.7 ± 5.6	0.556
PPG post TIPS (mmHg), mean ± SD	7.2 ± 2.7	6.9 ± 3.2	0.507
Reduction of PPG (mmHg), mean ± SD	12.2 ± 4.9	11.9 ± 5.1	0.741
Reduction of PPG (%), mean ± SD	62.0 ± 14.8	63.2 ± 14.1	0.605
BL bilirubin (mg/dl), mean ± SD	1.45 ± 0.98	1.55 ± 1.22	0.524
BL creatinine (mg/dl), mean ± SD	1.22 ± 0.66	1.26 ± 0.67	0.736
BL INR, mean ± SD	1.27 ± 0.18	1.28 ± 0.19	0.700
BL platelets (G/L), mean ± SD	149 ± 72	166 ± 90	0.158
BL sodium (mmol/L), mean ± SD	135 ± 4	133 ± 5	0.013
BL albumin (g/dl), mean ± SD	32 ± 6	31 ± 7	0.235
BL AST (U/L), mean ± SD	42 ± 23	49 ± 31	0.124
BL ALT (U/L), mean ± SD	26 ± 16	30 ± 22	0.216
BL white blood cell count (G/L), mean ± SD	6.7 ± 2.8	7.0 ± 3.3	0.554
BL intake of diuretics, n (%)	87 (88.8)	77 (96.3)	0.065
Spironolactone dose (mg per day), median (IQR)	150 (50–200)	100 (50–200)	0.249
Furosemide dose (mg per day), median (IQR)	40 (6–80)	80 (40–120)	0.014
**Characteristics at 3M**			
3M MELD score, mean ± SD	14± 5	14 ± 5	0.635
3M FIPS, mean ± SD	-0.24 ± 0.8	-0.26 ± 0.8	0.910
3M bilirubin (mg/dl), mean ± SD	2.4 ± 3.0	2.5 ± 3.3	0.954
3M creatinine (mg/dl), mean ± SD	1.0 ± 0.5	1.05 ± 0.5	0.698
3M INR, mean ± SD	1.3 ± 0.2	1.4 ± 0.4	0.300
3M platelets ($10^3/\mu l$), mean ± SD	132 ± 56	147 ± 77	0.119
3M sodium (mg/dl), mean ± SD	137 ± 4	136 ± 5	0.359
3M albumin (g/L), mean ± SD	32 ± 5	30 ± 5	0.028
3M AST (U/L), mean ± SD	57 ± 50	49 ± 31	0.267
3M ALT (U/L), mean ± SD	33 ± 22	37 ± 66	0.520
3M white blood cell count ($10^3/\mu l$), mean ± SD	7.1 ± 4.1	7.1 ± 4.9	0.994
3M intake of diuretics, n (%)	88 (87.1)	65 (81.1)	0.277
3M spironolactone dose (mg per day), median (IQR)	100 (50–150)	100 (0–188)	0.927
3M furosemide dose (mg per day), median (IQR)	20 (0–40)	40 (0–80)	0.133

Categorial variables were compared using the chi-squared test or Fisher's exact test, as appropriate. Continuous variables were compared using Mann-Whitney U test or an unpaired t-test, as appropriate.
Therefore, the summation of percentages may exceed 100%. 3M, 3 months after TIPS placement; ALD, alcohol-related liver disease; ALT, alanine aminotransferase; AST, aspartate aminotransferase; BL, baseline at the time of TIPS insertion; FIPS, Freiburg Index of Post-TIPS Survival; INR, International normalized ratio; MASH, metabolic dysfunction-associated steatohepatitis; MELD, Model for End-Stage Liver Disease; PH, portal hypertension; PPG, portal pressure gradient; TIPS, transjugular intrahepatic portosystemic shunt.
*Some patients had mixed TIPS indication and/or etiology of liver cirrhosis.

medication between patients with no *vs.* minimal ascites. At 3M, 88 (87.1%) patients in the no ascites group were treated with diuretics compared with 65 (81.1%) in the minimal ascites group (*p* = 0.277). Remarkably, the median furosemide dose of patients with minimal ascites was significantly higher compared with patients with no ascites at TIPS insertion (no ascites, 40 (6–80) mg per day; minimal ascites, 80 (40–120) mg per day, *p* = 0.014). However, this discrepancy did not persist at 3M.

Clinical outcome and follow-up

Overall, the median follow-up time was 678 days. Patients without residual ascites at 3M had the longest median follow-up duration (no ascites: 1,045 [IQR 439–2,076] days *vs.* minimal ascites: 788 [IQR 445–1,441] days *vs.* moderate/severe ascites: 518 [IQR 214–929] days; *p* <0.001). In addition, 44.5% (n = 130) experienced further decompensation and 50.7% (n = 148) of patients died. The distribution of different decompensating

Table 3. Cumulative incidence rates of further decompensation and transplant-free mortality in patients with advanced chronic liver disease with no ascites, minimal ascites, and moderate/severe ascites at 3 months after transjugular intrahepatic portosystemic shunt implantation considering liver transplantation and death (if appropriate) as a competing event utilizing Gray's test (Analysis 1).

		Cumulative incidence rate			
**Clinical outcome**	**Parameter**	**1 year**	**2 years**	**3 years**	***p* value**
Further decompensation	No ascites	13.6%	26.9%	31.9%	0.044
	Minimal ascites	33.3%	42.0%	48.9%	—
	Moderate/severe ascites	33.6%	39.6%	45.6%	—
Transplant-free mortality	No ascites	8.6%	13.4%	18.6%	0.007
	Minimal ascites	9.0%	11.9%	30.6%	—
	Moderate/severe ascites	22.4%	36.6%	48.8%	—

events is shown in Table S3. Furthermore, 14.2% (n = 42) of patients underwent LTx and 8.2% of patients (n = 24) developed hepatocellular carcinoma after TIPS insertion.

Analysis 1: clinical outcomes of patients with no ascites vs. minimal ascites vs. moderate/severe ascites at 3M

Importantly, the cumulative incidence of transplant-free mortality was significantly lower in patients with no residual ascites after TIPS implantation, whereas the highest transplant-free mortality rates were observed in patients with moderate/severe ascites (at 3 years of follow-up: no ascites, 18.6% *vs.* minimal ascites, 30.6% *vs.* moderate/severe ascites, 48.8%; $p = 0.007$; Table 3 and Fig. 2). At the same time, there was no statistically significant difference in the cumulative incidence of LTx (at 3 years of follow-up: no ascites, 12.7% *vs.* minimal ascites, 7.1% *vs.* moderate/severe ascites: 17.9%; $p = 0.169$).

Moreover, patients with moderate/severe ascites and patients with minimal ascites exhibited a similar cumulative incidence of further decompensation, which was considerably higher than in patients with no ascites (at 3 years of follow-up: no ascites, 31.9% *vs.* minimal ascites, 48.9%; *vs.* moderate/severe ascites, 45.6%; $p = 0.044$; Fig. S1).

Analysis 2: Clinical outcomes of patients with minimal ascites vs. no ascites at 3M after TIPS insertion

Patients with minimal ascites at 3M experienced significantly higher rates of further decompensation compared with patients with no ascites (subdistribution hazard ratio [SHR], 1.76; 95% CI, 1.14–2.73; $p = 0.011$; Fig. 3 and Table 4). This result stayed consistent after adjusting for removed primary etiological factor at 3M, MELD at 3M, and albumin at 3M (adjusted SHR [aSHR], 1.69; 95% CI, 1.03–2.77; $p = 0.038$). Applying a different model adjusting for removed primary etiological factor at 3M, FIPS at 3M, and albumin at 3M yielded similar results (aSHR, 1.76; 95% CI, 1.07–2.88; $p = 0.026$). Minimal ascites was not associated with hepatic encephalopathy (Table S5).

Importantly, minimal ascites at 3M cement was also associated with a significantly higher transplant-free mortality in univariable analysis (SHR, 1.69; 95% CI, 1.13–2.53; $p = 0.011$; Fig. 3). This finding stayed consistent after applying two different multivariate competing risk regression models (Adjusted model I: aSHR, 1.76; 95% CI, 1.08–2.88; $p = 0.024$; Adjusted model II: aSHR, 1.73; 95% CI, 1.05–2.82; $p = 0.030$). Only minimal ascites remained associated with transplant-free mortality in either multivariable model. Further details are provided in Table 4.

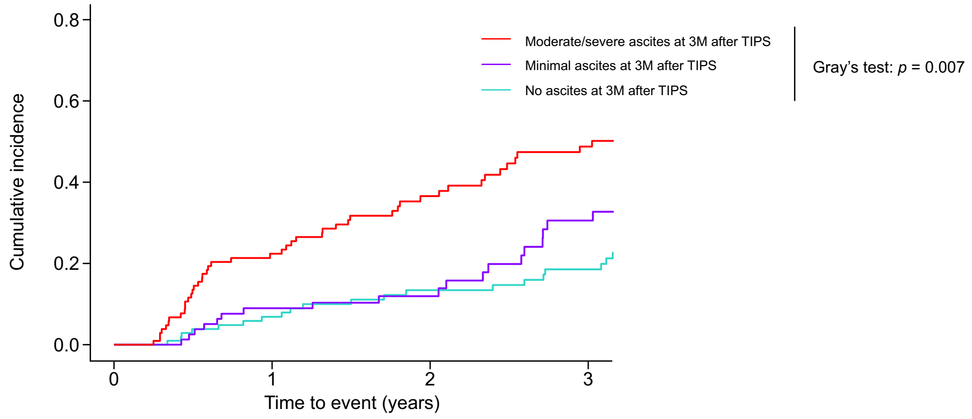

No. of patients at risk				
Moderate/severe ascites at 3M after TIPS	105	62	35	24
Minimal ascites at 3M after TIPS	82	63	43	29
No ascites at 3M after TIPS	105	84	64	51

Fig. 2. Cumulative incidence of transplant-free mortality in patients with no ascites, minimal ascites, and moderate/severe ascites 3 months after TIPS implantation. Liver transplantation was considered as a competing event. Cumulative incidences were compared using Grey's test. Levels of significance: $p = 0.007$. 3M, 3 months after TIPS insertion; TIPS, transjugular intrahepatic portosystemic shunt.

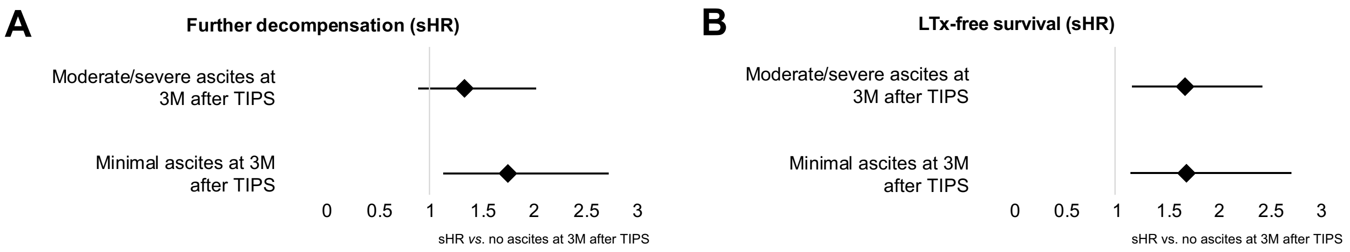

Fig. 3. sHR of univariable competing risk analysis compared with no ascites at 3M. (A) Further decompensation (sHR): moderate/severe ascites–minimal ascites at 3M, sHR *vs.* no ascites at 3M. (B) Death/LTx (sHR): moderate/severe ascites–minimal ascites at 3M, sHR *vs.* no ascites at 3M. 3M, 3 months after TIPS insertion; LTx, liver transplantation; sHR, subdistribution hazard ratio; TIPS, transjugular intrahepatic portosystemic shunt.

Discussion

This study thoroughly investigated the impact of residual ascites on the prognosis of patients 3 months after TIPS implantation. In our large retrospective cohort of patients with ACLD undergoing TIPS implantation for ascites in Vienna and Hannover, we demonstrated that the presence of ascites at 3M was linked to impaired clinical outcomes. First, patients with residual moderate/severe ascites exhibited a particularly poor prognosis, with approximately one-third experiencing further decompensation and a quarter dying within 15 months after TIPS implantation. Second, even minimal ascites at 3M (*i.e.* ascites that could only be detected via imaging) was an independent risk factor for further decompensation and mortality compared with patients without ascites at this time point, highlighting the clinical relevance of any residual ascites after TIPS implantation.

The onset of ascites is a hallmark in the natural history of ACLD and associated with a worsening of prognosis and quality of life.[1] Invention of the TIPS revolutionized the treatment of PH-derived complications, including recurrent and refractory ascites.[9] Although TIPS insertion can lead to ascites resolution, some patients still suffer from ascites, even months after the intervention.[11] Specifically, in our cohort, 64% of patients still exhibited some degree of ascites at 3M. Of note, the prevalence of minimal ascites at 3M was 28% in our cohort. Our results are in line with those of other studies: Piecha *et al.* recently described ascites persistence after TIPS implantation with need for paracentesis beyond 3 months after the intervention in 26% of patients, whereas another study observed minimal ascites in 29% of patients at 6 weeks after TIPS placement.[11,14] These findings underscore the importance of assessing residual ascites (which might not be clinically apparent) and investigating the optimal and potential intensified management strategies in patients with residual ascites.

Indeed, our data indicate that persistence of any ascites at 3M is associated with a higher risk of further decompensation and death. This is in line with published literature regarding the relevance of severe ascites following TIPS insertion.[14] Queck *et al.* investigated the relevance of ascites after TIPS insertion

Table 4. Impact of minimal *vs.* no ascites 3 months after transjugular intrahepatic portosystemic shunt implantation on the risk of further decompensation and death (Analysis 2).

	Univariate (unadjusted) analysis			Adjusted model I			Adjusted model II		
**Parameter of interest**	**sHR**	**95% CI**	***p* value**	**asHR**	**95% CI**	***p* value**	**asHR**	**95% CI**	***p* value**
**Further decompensation**									
Minimal ascites, yes	1.76	1.14–2.73	0.011	1.69	1.03–2.77	0.038	1.76	1.07–2.88	0.026
Age, years	1.00	0.98–1.02	0.890	—	—	—	—	—	—
Sex (female)	0.90	0.56–1.43	0.650	—	—	—	—	—	—
Removed primary etiological factor at 3M, yes	0.69	0.44–1.08	0.110	0.72	0.44–1.19	0.200	0.71	0.44–1.16	0.170
MELD at 3M, points	1.07	1.02–1.12	0.010	1.03	0.98–1.09	0.230	—	—	—
FIPS at 3M, points	1.38	1.00–1.90	0.047	—	—	—	1.29	0.94–1.77	0.110
Albumin at 3M, g × L^{-1}	0.93	0.89–0.98	0.003	0.96	0.92–1.00	0.076	0.96	0.92–1.00	0.080
Sodium at 3M, mmol × L^{-1}	1.02	0.97–1.06	0.440	—	—	—	—	—	—
PPG reduction relative, %	0.57	0.21–1.51	0.260	—	—	—	—	—	—
Diuretics at 3M, yes	0.63	0.31–1.27	0.190	—	—	—	—	—	—
**Death**									
Minimal ascites, yes	1.69	1.13–2.53	0.011	1.76	1.08–2.88	0.024	1.73	1.05–2.82	0.030
Age, years	1.03	1.01–1.05	0.012	1.02	0.99–1.05	0.150	—	—	—
Sex (female)	1.06	0.68–1.66	0.790	—	—	—	—	—	—
Removed primary etiological factor at 3M, yes	0.69	0.45–1.03	0.070	0.71	0.45–1.12	0.150	0.69	0.44–1.08	0.110
MELD at 3M, points	1.03	0.99–1.07	0.110	1.01	0.97–1.06	0.560	—	—	—
FIPS at 3M, points	1.10	0.81–1.50	0.520	—	—	—	1.12	0.82–1.48	0.490
Albumin at 3M, g × L^{-1}	0.99	0.95–1.03	0.690	—	—	—	—	—	—
Sodium at 3M, mmol × L^{-1}	0.96	0.90–1.01	0.130	—	—	—	—	—	—
PPG reduction relative, %	0.21	0.04–0.99	0.049	0.45	0.11–1.85	0.270	0.40	0.11–1.48	0.170
Diuretics at 3M, yes	1.08	0.18–6.45	0.930	—	—	—	—	—	—

Univariable and two multivariable competing risk regression models are shown. Adjusted model I included MELD at 3M as a parameter of liver function, whereas Adjusted model II included FIPS at 3M. Liver transplantation and death were considered as competing risks for further decompensation, whereas liver transplantation was considered as a competing risk for death. 3M, 3 months after TIPS placement; aSHR: adjusted subdistribution hazard ratio; FIPS, Freiburg Index of Post-TIPS Survival; MELD, Model for End-Stage Liver Disease; PPG, portal pressure gradient; SHR, subdistribution hazard ratio; TIPS, transjugular intrahepatic portosystemic shunt.

and only observed a numerically altered survival comparing patients with minimal to those without ascites.[14] However, the focus of previous work was mainly on the relevance of PPG decrease on the clinical course after TIPS implantation and there was no multivariable competing risk analysis to investigate the impact of minimal ascites. Moreover, in our study, pre-/post-TIPS PPG and PPG reduction did not differ between patients with minimal ascites and those without ascites. Hence, the observed effect does not appear to be exclusively explained by the portal pressure decrease alone. Another important difference between the present study and that by Queck *et al.*[14] is that our cut-off for residual ascites assessment was at 3M compared with 6 weeks post TIPS in the study by Queck *et al.*

Notably, one previous study investigating the impact of minimal ascites in outpatients with ACLD demonstrated that patients with minimal ascites had a significantly lower survival compared with those without ascites.[17] Similarly, our study revealed that the presence of minimal ascites at 3M was independently linked to mortality. Furthermore, minimal ascites at 3M was a risk factor for further decompensation independent of age, MELD, and other parameters associated with worse prognosis in ACLD (*i.e.* albumin and sodium). This represents the most significant novel finding of this study and underlines the prognostic relevance of residual ascites in patients with ACLD after TIPS implantation.

Of note, in our study, patient characteristics between those with no ascites and with minimal ascites were overall comparable at 3M (and at the time of TIPS placement). Reduction of PPG, MELD, white blood cells, or sodium did not differ between the groups. Only albumin was lower in those with minimal residual ascites at 3M. Nevertheless, patient outcomes did differ significantly in terms of survival and further decompensation. Although this has never been described for patients with minimal ascites, it is consistent with a previous study that did not observe significant differences in pre-TIPS baseline parameters between patients with severe ascites compared with patients with no residual ascites after TIPS implantation despite thorough work-up.[11]

This is important, because assuming that the pathophysiological driving forces behind minimal ascites are the same or similar as in moderate/severe ascites, a difference in liver disease severity, resulting in increased systemic inflammation and vasodilatation, could have been expected. However, this was not the case in this study, indicating successful ascites mobilization following TIPS insertion not solely as a linear consequence of pre-TIPS baseline conditions. Interestingly, the intake of diuretics and corresponding doses were comparable between all groups in our study at 3M, suggesting the presence of minimal ascites was not merely a marker of insufficient diuretic therapy.

One of the main focus points in TIPS research is patient selection and risk stratification.[18,19] Given that some patients experience decompensation after TIPS insertion, the objective has been to anticipate patients at risk and thereby minimizing peri- and post-interventional complications.[20–22] This work represents a different approach to the same objective: although patients in this study had a pre-TIPS work-up, including a cardiac work-up and computed tomography, and, thus, were preselected, this work shows that applied risk stratification after the intervention can help to identify patients at a higher risk of further complications in the follow-up, irrespective of the post-TIPS PPG. A dynamic approach enables more individualized clinical care, potentially closing an important gap in the clinical course of patients and providing clinicians with tools needed to improve patient care during the post-TIPS period.

Historically, one method to determine a patient's risk status has been based on the need for paracentesis or the persistence of clinically overt ascites, leaving ~70% of patients who do not require paracentesis at 3M in a prognostic gray zone.[11] Our study divides 30% of patients from that group (patients with ascites only visible on ultrasound, not requiring intervention; *i.e.* minimal or Grade 1 ascites) and highlights their need for more attentive care during the post-TIPS period compared with patients with no ascites at 3M.

This study has several important limitations. First, the retrospective nature of this study limited specific data acquisitions (*e.g.* number of previous paracenteses) and could have led to some complications being missed or the introduction of a selection bias, even though both centers have a dedicated follow-up for all patients. Given missing clinical/imaging data, previous LTx, hepatic/extrahepatic malignancy, no available ultrasound at 3M, or follow-up <90 days, a relevant number of patients had to be excluded. However, key disease severity parameters (*i.e.* MELD and PPG) of excluded patients did not differ compared with the included population. Furthermore, patients presented with different sets of available data, obstructing the assessment of markers of systemic inflammation (*i.e.* C-reactive protein and IL-6) and cardiac function (*i.e.* echocardiography and N-terminal prohormone of brain natriuretic peptide). Furthermore, repeated PPG measurements post-TIPS placement were not available in all patients from either centers and, thus, could not be utilized for this study. The PPG values given in this study were measured under general anesthesia and, therefore, could have been different under awake conditions. However, all PPG measurements were performed under similar conditions, resulting in retained comparability of the measurements.

In conclusion, this study demonstrates the prognostic relevance of minimal ascites 3 months after TIPS implantation. Patients with moderate/severe ascites had the worst prognosis and, thus, should be evaluated for LTx as quickly as possible. Future studies on the therapeutic potential of a more proactive or aggressive treatment of residual ascites for a patient's prognosis and incidence of complications would also be of interest.

Affiliations

[1]*Department of Gastroenterology, Hepatology, Infectious Diseases and Endocrinology, Hannover Medical School, Hannover, Germany;* [2]*Division of Gastroenterology and Hepatology, Department of Medicine III, Medical University of Vienna, Vienna, Austria;* [3]*Vienna Hepatic Hemodynamic Lab, Division of Gastroenterology and Hepatology, Department of Medicine III, Medical University of Vienna, Vienna, Austria;* [4]*German Center for Infection Research (DZIF), Hannover/Braunschweig, Germany;* [5]*Department of Diagnostic and Interventional Radiology, Hannover Medical School, Hannover, Germany;* [6]*Division of Interventional Radiology, Department of Radiology, Medical University of Vienna, Vienna, Austria;* [7]*Christian Doppler Lab for Portal Hypertension and Liver Fibrosis, Medical University of Vienna, Vienna, Austria*

Abbreviations

3M, 3 months after TIPS insertion; ACLD, advanced chronic liver disease; ALD, alcohol-related liver disease; ALT, alanine aminotransferase; aSHR, adjusted subdistribution hazard ratio; AST, aspartate aminotransferase; BL, baseline at the time of TIPS insertion; FIPS, Freiburg Index of Post-TIPS Survival; HE, hepatic encephalopathy; HRS-AKI, hepatorenal syndrome - acute kidney injury; INR, international normalized ratio; LTx, liver transplantation; MASH, metabolic dysfunction-associated steatohepatitis; MELD, Model for End-Stage Liver Disease; PH, portal hypertension PPG, portal pressure gradient; PTFE, polytetrafluoroethylene; SBP, spontaneous bacterial peritonitis; SHR, subdistribution hazard ratio; TIPS, transjugular intrahepatic portosystemic shunt.

Financial support

Jim B. Mauz, Sarah L. Schütte, Anja Tiede and Hannah Rieland were supported by the "KlinStrucMed" program of the Hannover Medical School, which reveived funding by the "Else-Kröner-Fresenius-Stiftung" until 2022.

Conflicts of interest

Given their role as Associate Editor of this journal, MM had no involvement in the peer-review of this article and had no access to information regarding its peer-review. Full responsibility for the editorial process for this article was delegated to the Co-Editor of the journal, Virginia Hernández-Gea. BM reports lecture and/or consultant fees from AbbVie, Astella, BMS, Falk, Fujirebio, Gilead, Luvos, MSD, Norgine, Roche, and W.L. Gore & Associates. He also received research support from Altona, EWIMED, Fujirebio, and Roche. MS received travel support from MSD, Sandoz, BMS, AbbVie, and Gilead, and speaking honoraria from BMS and Gilead. MT received research grants, travel grants, speaker fees, and advised for Gilead Sciences; received consultancy fees from AbbVie, Albireo, Agomab, BiomX, Boehringer Ingelheim, Chemomab, Falk, Glaxo Smith Kline, Genfit, Hightide, Intercept, Ipsen, Jannsen, Mirum, MSD, Novartis, Pliant, Regulus, Siemens, and Shire; research funding from Albireo, Alnylam, Cymabay, Falk, Intercept, MSD, Takeda, and UltraGenyx; travel grants from AbbVie, Falk, Intercept, and Jannsen; speaker fees from Albireo, BMS, Falk, Intercept, Ipsen, MSD, and Madrigal; the Medical Universities of Graz and Vienna have filed patents on medical use of NorUDCA, on which MT is listed as a co-inventor. HW has received fees for lectures and/or consultations from AbbVie, Aligos, Altimmune, Biotest, BMS, BTG, Dicerna, Enanta, Gilead, Janssen, Merck/MSD, MYR GmbH, Roche, and Vir Biotechnology. TR served as a speaker and/or consultant and/or advisory board member for AbbVie, Bayer, Boehringer Ingelheim, Gilead, Intercept, MSD, Siemens, and W.L. Gore & Associates, and received grants/research support from AbbVie, Boehringer Ingelheim, Gilead, Intercept, MSD, Myr Pharmaceuticals, Pliant, Philips, Siemens, and W.L. Gore & Associates, as well as travel support from AbbVie, Boehringer Ingelheim, Gilead, and Roche. JM and LH declare no conflicts of interest.

Please refer to the accompanying ICMJE disclosure forms for further details.

Authors' contributions

Contributed to research design: JBM, LH, TR, TLT. Contributed to data acquisition: JBM, LH, AK, SLS, PH, TMB, MJ, AT, HR, MS, ND, GK. Contributed to data analysis: JBM, LH, TR, TLT. Contributed to data interpretation: all authors. Drafted the manuscript: JBM, LH, TR, TLT. Critically revised the manuscript: all other authors. Funding acquisition: MT, HW, MM, BM, TR.

Data availability statement

The data enabling the findings of this study are available from the corresponding author upon reasonable request.

Supplementary data

Supplementary data to this article can be found online at https://doi.org/10.1016/j.jhepr.2025.101335.

References

Author names in bold designate shared co-first authorship

[1] D'Amico G, Bernardi M, Angeli P. Towards a new definition of decompensated cirrhosis. J Hepatol 2022;76:202–207.
[2] **Balcar L**, **Tonon M**, Semmler G, Calvino V, et al. Risk of further decompensation/mortality in patients with cirrhosis and ascites as the first single decompensation event. JHEP Rep 2022;4:100513.
[3] Wong F. Innovative approaches to the management of ascites in cirrhosis. JHEP Rep 2023;5:100749.
[4] Reiberger T, Hofer BS. The Baveno VII concept of cirrhosis recompensation. Dig Liver Dis 2023;55:431–441.
[5] Hofer BS, Simbrunner B, Hartl L, et al. Hepatic recompensation according to Baveno VII criteria is linked to a significant survival benefit in decompensated alcohol-related cirrhosis. Liver Int 2023;43:2220–2231.
[6] Nadim MK, Kellum JA, Forni L, et al. Acute kidney injury in patients with cirrhosis: acute Disease Quality Initiative (ADQI) and International Club of Ascites (ICA) joint multidisciplinary consensus meeting. J Hepatol 2024;81:163–183.
[7] de Franchis R, Bosch J, Garcia-Tsao G, et al. Baveno VII Faculty. Baveno VII - renewing consensus in portal hypertension. J Hepatol 2022;76:959–974.
[8] Campbell MS, Brensinger CM, Sanyal AJ, et al. Quality of life in refractory ascites: transjugular intrahepatic portal-systemic shunting versus medical therapy. Hepatology 2005;42:635–640.
[9] Bureau C, Thabut D, Oberti F, et al. Transjugular intrahepatic portosystemic shunts with covered stents increase transplant-free survival of patients with cirrhosis and recurrent ascites. Gastroenterology 2017;152:157–163.
[10] Bucsics T, Hoffman S, Grünberger J, et al. ePTFE-TIPS *vs.* repetitive LVP plus albumin for the treatment of refractory ascites in patients with cirrhosis. Liver Int 2018;38:1036–1044.
[11] Piecha F, Radunski UK, Ozga A, et al. Ascites control by TIPS is more successful in patients with a lower paracentesis frequency and is associated with improved survival. JHEP Rep 2019;1:90–98.
[12] European Association for the Study of the Liver. European Association for the Study of the Liver. EASL Clinical Practice Guidelines for the management of patients with decompensated cirrhosis. J Hepatol 2018;69:406–460.
[13] Theodorakopoulos T, Kalafateli M, Kalambokis GN, et al. Natural history of grade 1 ascites in patients with liver cirrhosis. Ann Gastroenterol 2021;34:93–103.
[14] Queck A, Schwierz L, Gu W, et al. Targeted decrease of portal hepatic pressure gradient improves ascites control after TIPS. Hepatology 2023;77:466–475.
[15] Fine JP, Gray RJ. A proportional hazards model for the subdistribution of a competing risk. J Am Stat Assoc 1999;94:496–509.
[16] Jachs M, Hartl L, Schaufler D, et al. Amelioration of systemic inflammation in advanced chronic liver disease upon beta-blocker therapy translates into improved clinical outcomes. Gut 2021;70:1758–1767.
[17] Tonon M, Piano S, Gambino CG, et al. Outcomes and mortality of Grade 1 ascites and recurrent ascites in patients with cirrhosis. Clin Gastroenterol Hepatol 2021;19:358–366.
[18] Bettinger D, Sturm L, Pfaff L, et al. Refining prediction of survival after TIPS with the novel Freiburg index of post-TIPS survival. J Hepatol 2021;74:1362–1372.
[19] Stockhoff L, Schneider H, Tergast TL, et al. Freiburg index of post-TIPS survival (FIPS) a valid prognostic score in patients with cirrhosis but also an advisor against TIPS? J Hepatol 2021;75:487–489.
[20] Rössle M. TIPS: 25 years later. J Hepatol 2013;59:1081–1093.
[21] Billey C, Billet S, Robic MA, et al. A prospective study identifying predictive factors of cardiac decompensation after transjugular intrahepatic portosystemic shunt: the Toulouse Algorithm. Hepatology 2019;70:1928–1941.
[22] Deltenre P, Zanetto A, Saltini D, et al. The role of transjugular intrahepatic portosystemic shunt in patients with cirrhosis and ascites: recent evolution and open questions. Hepatology 2023;77:640–658.

Keywords: Cirrhosis; Portal hypertension; Transjugular intrahepatic portosystemic shunt; Ascites.
Received 6 July 2024; received in revised form 9 January 2025; accepted 17 January 2025; Available online 23 January 2025

Journal of Hepatology, Volume *7*

Supplemental information

Minimal residual ascites 3 months after TIPS implantation implicates worse clinical outcomes in patients with cirrhosis

Jim Benjamin Mauz, Lukas Hartl, Andrea Kornfehl, Sarah Lisa Schütte, Paul Hemetsberger, Theresa Müllner-Bucsics, Mathias Jachs, Anja Tiede, Hannah Rieland, Michael Schwarz, Nina Dominik, Georg Kramer, Bernhard Meyer, Lukas Reider, Michael Trauner, Heiner Wedemeyer, Mattias Mandorfer, Benjamin Maasoumy, Thomas Reiberger, and Tammo Lambert Tergast

Minimal residual ascites 3 months after TIPS implantation predicts worse clinical outcomes in patients with cirrhosis

Jim Benjamin Mauz, Lukas Hartl, Andrea Kornfehl, Sarah Lisa Schütte, Paul Hemetsberger, Theresa Müllner-Bucsics, Mathias Jachs, Anja Tiede, Hannah Rieland, Michael Schwarz, Nina Dominik, Georg Kramer, Bernhard Meyer, Lukas Reider, Michael Trauner, Heiner Wedemeyer, Mattias Mandorfer, Benjamin Maasoumy, Thomas Reiberger, Tammo Lambert Tergast

Table of contents

Supplementary Results……………………………………………………………….……......2

Fig. S1……………………………………………………………………………………….....3

Table S1……………………………………………………………………………………....4

Table S2……………………………………………………………………………………....5

Table S3………………………………………………………………………………………8

Table S4……………………………………………………………………………………...9

Table S5……………………………………………………………………………………..11

Supplementary Results

Patient characteristics of excluded patients (Table S1)

Table S1 depicts the key baseline characteristics of patients receiving TIPS for ascites who had to be excluded from the study (due to lack of clinical data, previous LTx, hepatic/extrahepatic malignancy, no available abdominal ultrasound at 3 months after TIPS implantation or follow-up <90 days) compared to the included population. Notably, MELD and PPG pre TIPS and relative PPG decrease did not differ between these two cohorts; while excluded patients were older and more commonly had viral etiology of cirrhosis.

Supplementary Figures

Fig. S1. Cumulative incidence of further decompensation in patients with no ascites, minimal ascites and moderate/severe ascites 3 months after TIPS implantation. Liver transplantation and death were considered as competing events. Cumulative incidences were compared using Grey's test.

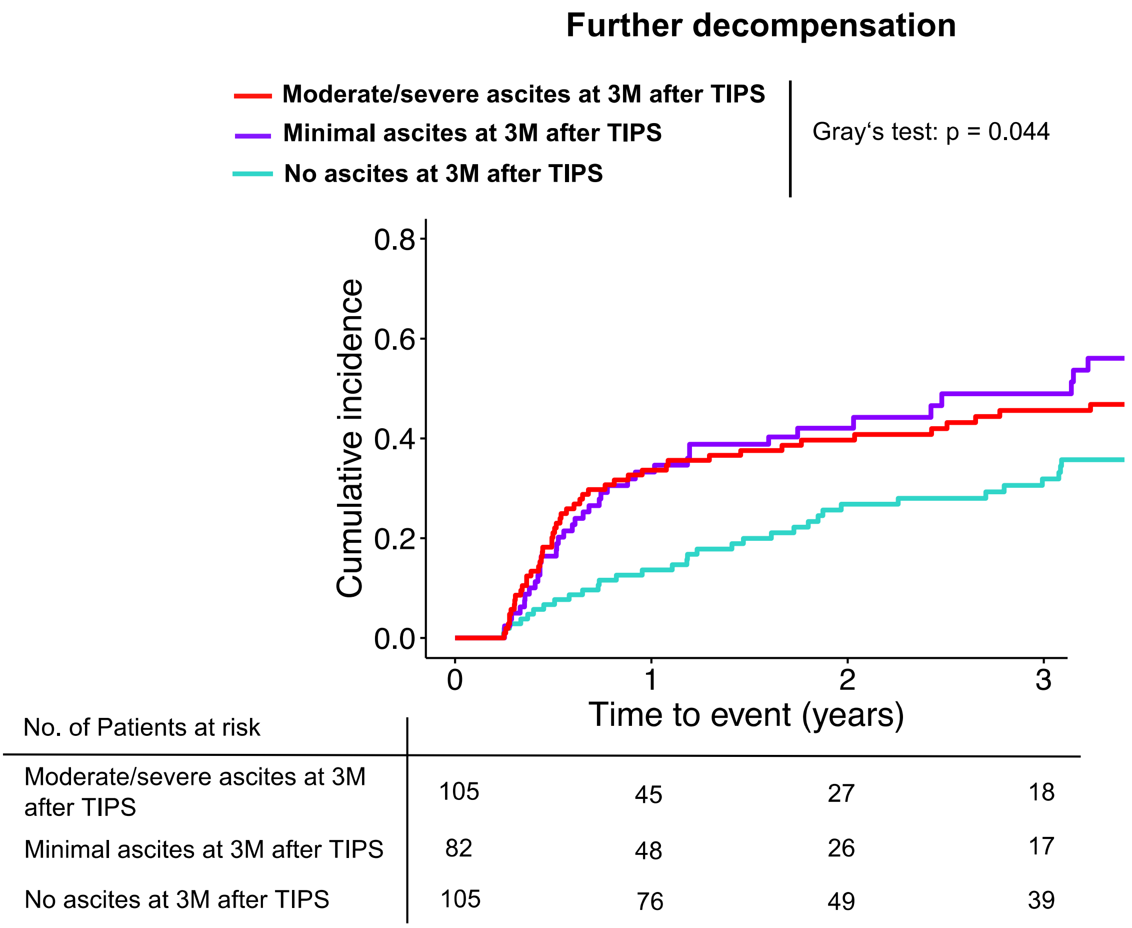

No. of Patients at risk				
Moderate/severe ascites at 3M after TIPS	105	45	27	18
Minimal ascites at 3M after TIPS	82	48	26	17
No ascites at 3M after TIPS	105	76	49	39

Abbreviations 3M: 3 months after TIPS insertion, TIPS: Transjugular intrahepatic portosystemic shunt

Supplementary Tables

Table S1. Baseline characteristics of patients who were included into the study and of patients with ACLD and TIPS implantation due to refractory ascites who were excluded due to lack of clinical data, hepatic/extrahepatic malignancy, previous liver transplantation, no available abdominal ultrasound at 3 months after TIPS implantation or follow-up <90 days.

**Characteristic**	**Included pts n=292**	**Excluded pts n=331**	**p-value**
**Sex: female,** n (%)	83 (28.4)	118 (35.6)	0.054
**Age (years),** mean ± SD	57.7 ± 10.1	59.6 ± 10.8	*0.004*
**Etiology of cirrhosis**[*]			
**ALD,** n (%)	209 (71.6)	129 (39.0)	*<0.001*
**MASH,** n (%)	9 (3.1)	21 (6.3)	0.058
**Viral,** n (%)	29 (9.9)	122 (36.9)	*<0.001*
**Cryptogenic,** n (%)	25 (8.6)	28 (8.5)	0.964
**Other**, n (%)	20 (6.8)	31 (9.4)	0.253
**MELD score,** mean ± SD	14 ± 5	14 ± 5	0.570
**FIPS,** mean ± SD	-0.21 ± 0.78	-0.06 ± 0.93	*0.040*
**PPG pre TIPS (mmHg),** mean ± SD	18.6 ± 5.5	17.8 ± 5.8	0.074
**PPG post TIPS (mmHg),** mean ± SD	7.2 ± 3.0	6.7 ± 3.3	*0.048*
**Reduction of PPG (%),** mean ± SD	60.5 ± 15.0	61.9 ± 16.1	0.300
**Bilirubin (mg/dL),** mean ± SD	1.42 ± 1.02	1.39 ± 1.44	0.749
**Creatinine (mg/dL),** mean ± SD	1.29 ± 0.75	1.40 ± 0.93	0.096
**INR,** mean ± SD	1.28 ± 0.19	1.29 ± 0.23	0.654
**Sodium (mmol/L),** mean ± SD	134 ± 5	134 ± 6	0.393
**Albumin (g/dL),** mean ± SD	32 ± 6	30 ± 7	*0.003*

[*]Some patients had mixed TIPS indication and/or etiology of liver cirrhosis. Therefore, the summation of percentages may exceed 100%.

Abbreviations ALD: Alcohol-related liver disease, FIPS: Freiburg index of post-TIPS survival, INR: International normalized ratio, MASH: Metabolic dysfunction-associated steatohepatitis, MELD: Model for end-stage liver disease, PPG: Portal pressure gradient, TIPS: Transjugular intrahepatic portosystemic shunt.

Table S2. Baseline characteristics: no ascites or minimal ascites vs. moderate/severe ascites at month 3 after TIPS insertion

Characteristic	No or minimal ascites n=187 (64.0)	Moderate/severe ascites n=105 (36.0)	p-value
**Sex: female**, n (%)	63 (33.7)	20 (19.0)	*0.008*
**Age (years),** mean ± SD	57.3 ± 9.8	58.4 ± 10.6	0.373
**Etiology of cirrhosis***			
**ALD,** n (%)	127 (67.9)	82 (78.1)	0.064
**MASH,** n (%)	5 (2.7)	4 (3.8)	0.727
**Viral,** n (%)	20 (10.7)	9 (8.6)	0.560
**Cryptogenic,** n (%)	18 (9.6)	7 (6.7)	0.386
**Other,** n (%)	17 (9.1)	3 (2.9)	*0.043*
**Main indication for TIPS***			
**Ascites**, n (%)	187 (100)	105 (100)	
**PH-related Bleeding,** n (%)	18 (9.6)	6 (5.7)	0.243
**Other**, n (%)	5 (2.7)	2 (1.9)	1.000
**BL MELD score**, mean ± SD	14 ± 5	14 ± 5	0.949
**BLChild Pugh**			
**Class A,** n (%)	0 (0.0)	0 (0.0)	
**Class B,** n (%)	150 (80.2)	91 (86.7)	0.199
**Class C,** n (%)	27 (14.4)	13 (12.4)	0.624
**BL FIPS,** mean ± SD	-0.22 ± 0.77	-0.18 ± 0.78	0.645
**PPG pre TIPS (mmHg),** mean ± SD	18.9 ± 5.4	18.1 ± 5.5	0.199
**PPG post TIPS (mmHg),** mean ± SD	7.0 ± 2.9	7.6 ± 3.1	0.166
**Reduction of PPG (mmHg),** mean ± SD	12.1 ± 5.0	10.6 ± 4.6	*0.016*
**Reduction of PPG (%),** mean ± SD	62.5 ± 14.5	57.2 ± 15.2	*0.005*
**BL Bilirubin (mg/dL),** mean ± SD	1.50 ± 1.09	1.29 ± 0.87	0.104
**BL Creatinine (mg/dL),** mean ± SD	1.24 ± 0.66	1.38 ± 0.88	0.135
**BL INR,** mean ± SD	1.28 ± 0.18	1.27 ± 0.22	0.817
**BL Platelets (10^3/µL),** mean ± SD	156 ± 80	164 ± 104	0.529
**BL Sodium (mmol/L),** mean ± SD	134 ± 5	134 ± 5	*0.465*

**BL Albumin (g/dL),** mean ± SD	32 ± 6	32 ± 5	0.830
**BL AST (U/L),** mean ± SD	45 ± 27	44 ± 23	0.790
**BL ALT (U/L),** mean ± SD	28 ± 19	26 ± 14	0.365
**BL White blood cell count (10^3/µL),** mean ± SD	6.8 ± 3.0	6.2 ± 3.4	0.127
**BL Intake of diuretics,** n (%)	164 (92.1)	94 (90.4)	0.611
**Spironolactone dose (mg per day),** median (IQR)	100 (50-200)	100 (0-200)	0.768
**Furosemide dose (mg per day),** median (IQR)	60 (20-80)	60 (40-100)	0.954
**Characteristics at month 3 after TIPS insertion (3M)**			
**3M MELD score,** mean ± SD	14 ± 5	16 ± 6	*0.034*
**3M FIPS,** mean ± SD	-0.25 ± 0.8	-0.17 ± 0.8	*0.025*
**3M Bilirubin (mg/dL),** mean ± SD	2.4 ± 3.2	2.2 ± 1.9	0.427
**3M Creatinine (mg/dL),** mean ± SD	1.0 ± 0.5	1.3 ± 1.0	*0.015*
**3M INR,** mean ± SD	1.4 ± 0.3	1.4 ± 0.2	0.687
**3M Platelets (10^3/µL),** mean ± SD	139 ± 66	145 ± 87	0.511
**3M Sodium (mg/dL),** mean ± SD	136 ± 5	135 ± 5	*0.049*
**3M Albumin (g/L),** mean ± SD	31 ± 5	30 ± 6	0.081
**3M AST (U/l),** mean ± SD	53 ± 43	50 ± 45	0.587
**3M ALT (U/l),** mean ± SD	34 ± 47	25 ± 17	0.056
**3M White blood cell count (10^3/µL),** mean ± SD	7.1 ± 4.4	7.0 ± 6.0	0.913
**3M Intake of diuretics**, n (%)	153 (84.5)	86 (83.5)	0.818
**3M Spironolactone dose (mg per day),** median (IQR)	100 (38-175)	100 (0-200)	0.256
**3M Furosemide dose (mg per day),** median (IQR)	40 (0-60)	40 (0-80)	0.924

*Some patients have mixed TIPS indication and/or etiology of liver cirrhosis. Therefore, the summation of percentages may exceed 100%.

Abbreviations ALD: Alcohol-related liver disease, ALT: Alanine aminotransferase, AST: Aspartate aminotransferase, BL: at the time of TIPS insertion, FIPS: Freiburg index of post-TIPS survival, INR: International normalized ratio, MASH: Metabolic dysfunction-associated steatohepatitis, MELD: Model for end-stage liver disease, 3M: 3 Months after TIPS placement,

PH: Portal hypertension, PPG: Portal pressure gradient, TIPS: Transjugular intrahepatic portosystemic shunt.

Table S3. Incidences of events

	No ascites n=105 (36.0%)	Minimal ascites n=82 (28.1%)	Moderate/severe ascites n=105 (36.0%)
**Death**	48 (45.7%)	41 (50.0%)	59 (56.2%)
**Liver transplantation**	15 (14.3%)	7 (8.5%)	20 (19.0%)
**Further decompensation***	41 (39.0%)	40 (48.8%)	49 (46.7%)
**Worsening of Ascites#**	10 (9.5%)	22 (26.8%)	25 (23.8%)
**Overt HE**	28 (26.7%)	22 (26.8%)	24 (22.9%)
**PH-bleeding**	2 (1.9%)	0 (0.0%)	1 (1.0%)
**SBP**	2 (1.9%)	0 (0.0%)	9 (8.6%)
**HRS-AKI**	2 (1.9%)	1 (1.2%)	6 (5.7%)

*Some patients experienced multiple decompensating events at the same time. Therefore, the summation of percentages may exceed 100%.

#Worsening of ascites compatible with further hepatic decompensation was defined as formation of ascites that required paracentesis, hospitalization and/or was associated with further complications (i.e., SBP, HRS-AKI)."

Abbreviations HE: Hepatic encephalopathy, HRS-AKI: Hepatorenal syndrome - acute kidney injury, PH: Portal hypertension, SBP: Spontaneous bacterial peritonitis.

Table S4. Patient characteristics: Hannover cohort vs. Vienna cohort

Characteristic	Hannover cohort n=84 (28.9%)	Vienna cohort n=208 (71.2%)	p-value
**Sex: female,** n (%)	25 (29.8)	58 (27.9)	0.747
**Age (years),** mean ± SD	56.6 ± 11.1	58.2 ± 9.7	0.278
**Etiology of cirrhosis***			
**ALD,** n (%)	52 (61.9)	157 (75.5)	*0.020*
**MASH,** n (%)	5 (6.0)	4 (1.9)	0.126
**Viral,** n (%)	6 (7.1)	23 (11.1)	0.311
**Cryptogenic,** n (%)	9 (10.7)	16 (7.7)	0.430
**Other**, n (%)	12 (14.3)	8 (3.8)	*0.001*
**Indication for TIPS***			
**Ascites**, n (%)	84 (100)	208 (100)	
**PH-related Bleeding,** n (%)	7 (8.3)	17 (8.2)	0.964
**BL MELD score,** mean ± SD	13 ± 4	14 ± 5	*0.011*
**BL Child Pugh**			
**Class A,** n (%)	0 (0.0)	0 (0.0)	
**Class B,** n (%)	76 (90.5)	176 (84.6)	0.187
**Class C,** n (%)	8 (9.5)	32 (15.4)	0.187
**BL FIPS,** mean ± SD	-0.18 ± 0.67	-0.22 ± 0.82	0.720
**Reduction of PPG (%),** mean ± SD	60.2 ± 15.7	60.7 ± 14.7	0.818
**Bilirubin (mg/dL),** mean ± SD	1.18 ± 1.03	1.51 ± 1.00	*0.010*
**Creatinine (mg/dL),** mean ± SD	1.41 ± 0.84	1.24 ± 0.71	0.081
**INR,** mean ± SD	1.28 ± 0.19	1.27 ± 0.20	0.978
**Platelets (G/L),** mean ± SD	146 ± 94	164 ± 88	0.127
**Sodium (mmol/L),** mean ± SD	134 ± 5	134 ± 5	0.470
**Albumin (g/dL),** mean ± SD	29 ± 7	33 ± 5	*<0.001*
**AST (U/L),** mean ± SD	51 ± 29	42 ± 24	*0.015*
**ALT (U/L),** mean ± SD	30 ± 23	25 ± 5	0.069
**White blood cell count (G/L),** mean ± SD	6.2 ± 3.2	6.7 ± 3.1	0.195
**Outcomes** at 2 years post-TIPS insertion			
**Death**, n (%)	12 (14.3)	47 (22.6)	0.109
**Liver Transplantation**, n (%)	9 (10.7)	24 (11.5)	0.840
**HCC development**, n (%)	6 (7.1)	2 (1)	*0.008*
**Decompensation**, n (%)	35 (41.7)	64 (30.8)	0.075

[*]Some patients have mixed TIPS indication and/or etiology of liver cirrhosis. Therefore, the summation of percentages may exceed 100%.

Abbreviations ALD: Alcohol-related liver disease, ALT: Alanine aminotransferase, AST: Aspartate aminotransferase, BL: at the time of TIPS insertion, FIPS: Freiburg index of post-TIPS survival, HE: Hepatic encephalopathy, INR: International normalized ratio, MASH: Metabolic dysfunction-associated steatohepatitis, MELD: Model for end-stage liver disease, PH: Portal hypertension, PPG: Portal pressure gradient, TIPS: Transjugular intrahepatic portosystemic shunt, 3M: 3 Months after TIPS placement.

Table S5. Impact of minimal versus no ascites 3 months after transjugular intrahepatic portosystemic shunt (TIPS) implantation on the risk of hepatic encephalopathy. Univariable and two multivariable competing risk regression models are shown. Adjusted model I included MELD at 3M as a parameter of liver function, while Adjusted model II included FIPS at 3M. Liver transplantation and death were considered as competing risks.

**Parameter of interest**	**Univariate (unadjusted) analysis**			**Adjusted model I**			**Adjusted model II**		
**hepatic encephalopathy**	**sHR**	**95%CI**	**p-value**	**asHR**	**95%CI**	**p-value**	**asHR**	**95%CI**	**p-value**
**Minimal ascites, yes**	1.18	0.75-1.85	0.470	1.42	0.87-2.33	0.160	1.39	0.85-2.29	0.190
**Age, year**	1.02	1.00-1.05	**0.037**	1.04	1.01-1.06	**0.017**	-	-	-
**Sex (female)**	0.76	0.46-1.24	0.270	-	-	-	-	-	-
**Removed primary etiological factor at 3M, yes**	0.81	0.52-1.28	0.360	0.81	0.50-1.32	0.400	0.85	0.53-1.37	0.510
**MELD at 3M, points**	1.06	1.01-1.11	**0.014**	1.05	1.00-1.11	**0.034**	-	-	-
**FIPS at 3M, points**	1.47	1.07-2.01	**0.018**	-	-	-	1.41	1.03-1.94	**0.034**
**Albumin at 3M, g x L^{-1}**	0.96	0.92-1.00	0.060	0.99	0.94-1.03	0.570	0.98	0.93-1.02	0.290
**Sodium at 3M, mmol x L^{-1}**	0.98	0.92-1.03	0.380	-	-	-	-	-	-
**PPG reduction relative, %**	1.32	0.22-7.96	0.760	-	-	-	-	-	-
**Diuretics at 3M, yes**	1.85	0.20-17.00	0.580	-	-	-	-	-	-

Abbreviations FIPS: Freiburg index of post-TIPS survival, MELD: Model for end-stage liver disease, 3M:3 Months after TIPS placement, PPG: Portal pressure gradient, TIPS: Transjugular intrahepatic portosystemic shunt
